# Superwarfarin Rodenticide Poisoning due to Consumption of Exotic Animals: A Case Report

**DOI:** 10.1155/crh/9981550

**Published:** 2025-11-17

**Authors:** Batoul Sadek, Cassandra Dasmarinas, Ishita Patel, Lynda Bowman, Peter Xie, Eric Irons, Albert Jang

**Affiliations:** ^1^Department of Internal Medicine-Pediatrics, Case Western University, Cleveland, Ohio, USA; ^2^Department of Internal Medicine, Case Western University, Cleveland, Ohio, USA; ^3^Department of Pathology, MetroHealth Hospital, Case Western University, Cleveland, Ohio, USA; ^4^Division of Hematology/Oncology, MetroHealth System, Case Western Reserve University, Cleveland, Ohio, USA; ^5^Divisions of Hematology and Solid Tumor Oncology, Department of Medicine, University Hospitals Cleveland Medical Center, Case Western Reserve University School of Medicine, Cleveland, Ohio, USA

**Keywords:** case report, rodenticide, superwarfarin, vitamin K

## Abstract

Poisoning from superwarfarin rodenticides should be considered in patients with unexplained bleeding due to vitamin K deficiency, with no other history of coagulopathy or anticoagulant use. A 37-year-old man originally presented to our hospital with two weeks of oral bleeding and two days of hematuria of unknown etiology. Workup showed severely prolonged prothrombin time and extremely low activity of coagulation factors II, VII, IX, and X, raising suspicion for vitamin K deficiency. His coagulation studies gradually corrected after daily administration of high-dose intravenous vitamin K. An anticoagulant poisoning panel ultimately revealed high levels of brodifacoum rodenticide—likely from rodent meat ingestion during his vacation to China 2 months before. Our case highlights the importance of a thorough social and toxicologic investigation in patients with unexplained coagulopathy consistent with significant vitamin K deficiency.

## 1. Introduction

Long-acting anticoagulant rodenticides (LAARs), also known as superwarfarins, are long-acting warfarin derivatives designed to eradicate rodents resistant to warfarin [1]. These compounds have the same mechanism of action as warfarin, inhibiting vitamin K epoxide reductase, thus preventing the carboxylation of vitamin K-dependent coagulation factors II, VII, IX, X, protein C, and protein S. This ultimately results in life-threatening hemorrhage [2]. Brodifacoum is one of the most widely used LAARs. It is known for its higher potency and longer half-life compared with other superwarfarin compounds, resulting in coagulopathy lasting from two to twelve months even after serum levels are undetectable. In cases of human ingestion, treatment relies on long-term administration of large daily doses of vitamin K (20–125 mg per day) [3].

Given its widespread use in rodenticides and a few documented cases of its detection in synthetic cannabinoids, most brodifacoum poisoning cases usually occur through accidental ingestion and/or exposure [4]. As such, poisoning from compounds like brodifacoum and other LAARs is a rare but important differential diagnosis to consider in the evaluation of patients presenting with symptoms of acute hemorrhage without previous coagulopathy or risk factors for bleeding. Following accidental exposure to LAARs, patients classically present with symptoms of mucocutaneous bleeding (e.g., menorrhagia, melena, epistaxis, or hematuria) without a history of anticoagulation or bleeding disorder [5]. Here, we discuss a case of a patient presenting with persistent oral bleeding and hematuria who was found to have poisoning by brodifacoum, likely due to ingestion of poisoned rodents in rural China.

## 2. Case Study

A 37-year-old Chinese man with a history of poorly controlled type 2 diabetes mellitus and chronic hepatitis B on tenofovir presented to the hospital for two weeks of intermittent gum and nose bleeding and two days of hematuria. He was seen by his primary care physician 2 weeks prior for these concerns and was urgently referred to gastroenterology to evaluate for a suspected bleeding upper gastrointestinal lesion. An esophagogastroduodenoscopy was performed without evidence of esophageal or gastric bleeding. Rather, bleeding was noted in the oral cavity. He was then referred to dentistry and was prescribed oral tranexamic acid for 2 days without improvement. He subsequently developed hematuria, prompting the hospital visit.

On physical exam, there was dried blood on his lips and left nare with some oozing from both these sites. On interview, he denied any illnesses or sick contacts with similar symptoms. He expressed concern that his bleeding may be related to alcohol consumption at a party several weeks prior. He had no history of previous coagulopathy and denied ever having mucosal bleeding, excessive bleeding from injuries or surgeries, or episodes of joint effusions and pain. He denied anticoagulant use. He reported active tobacco use, occasional alcohol use, and a diet consisting mostly of leftover meats with trace amounts of vegetables and fruits. He was an immigrant from China who had lived in the United States for 15 years, working as a chef in a Chinese restaurant, and reported recently returning from a month-long trip to China 2 months prior. Family history was significant for systemic lupus erythematosus (SLE) in his sister, with whom he lived. There was no family history of bleeding disorders.

**Figure 1:**
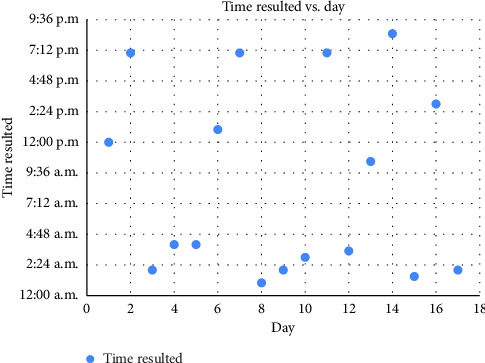
Time and day of vitamin K administration.

**Figure 2:**
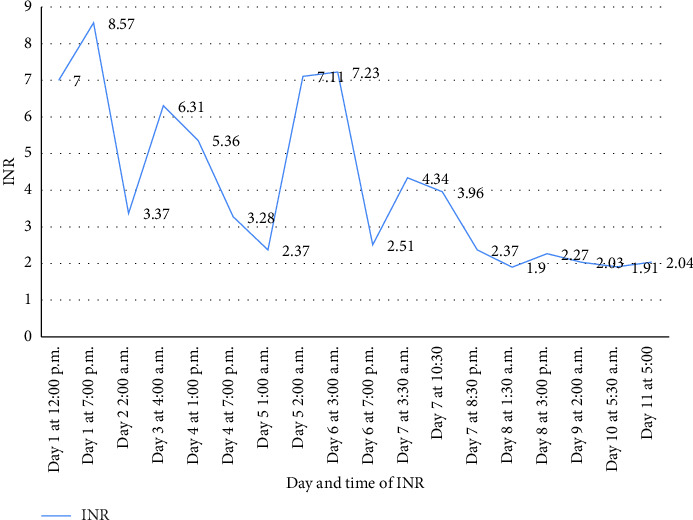
INR values during stay.

The patient had a blood pressure of 106/71, pulse of 86, temperature of 36.6°C, respiratory rate of 16, and SpO2 of 96%. Initial workup was significant for white blood cell count of 6.6 K/μL, hemoglobin of 7.2 g/dL, platelet count of 226 K/μL, prothrombin time (PT) of 112 s with incalculably high international normalized ratio (INR), a partial thromboplastin time of 112 s, fibrinogen of 435 mg/dL, and D-dimer of < 200 ng/mL. Coagulation factor activity assays revealed low levels of vitamin K-dependent factors II (4%), VII (< 1%), IX (5%), and X (1%) and preserved factor V (75%). This raised concern for vitamin K deficiency, and he was initially treated with four intravenous (IV) doses of 10 mg vitamin K over 48 h. The INR improved following the third dose but again increased before the fourth dose was given. The timeline of vitamin K administration times and INR results throughout his hospitalization is detailed in Figure1. Dates and times of administration of vitamin K are shown in Figure 2. INR measurements and serial measurements of the patient's prothrombin time and activated partial thromboplastin time are shown since day of admission, along with times of administration of intravenous vitamin K.

Since his coagulation panel demonstrated a definite yet incomplete response to vitamin K supplementation, with corresponding resolution of all bleeding, a diagnosis of vitamin K deficiency was made. However, given the lack of warfarin or other anticoagulant use, the cause of this remained unclear, and exposure to LAARs was considered. The patient adamantly denied use of rodenticide either at his place of employment or at home, stating that there had been no concerns with rodent infestation. He further denied any family members using warfarin. An anticoagulant poisoning panel was sent for further investigation. Results were positive for brodifacoum (qualitative). Given the lack of plausible exposure, suicidal attempt or intentional poisoning became possibilities. On further questioning, however, the patient recalled consumption of rodents and other exotic animals at a street market in China during his vacation.

He was treated with 13 more doses of 10 mg IV vitamin K throughout his hospitalization. aPTT normalized and INR gradually decreased to 2.04 at discharge. He was discharged with an oral dose of 100 mg daily vitamin K and continues to follow up with hematology for INR and symptomatic monitoring. In the outpatient setting, the INR continued to be labile over the next 3 months, ranging from 1.00 to 1.91. Four months after discharge, his INR was 0.94 while taking oral vitamin K 100 mg daily. To date, he has experienced no further episodes of bleeding.

## 3. Discussion

In the United States, approximately 10,400 cases of rat poisoning occur per year [2]. Exposure to rodenticides occurs primarily in children, who account for 90% of ingestions [2], individuals with psychiatric conditions [6], and individuals who use cannabinoids that are contaminated with rodenticide [4]. Most adult cases are due to accidental exposure to contaminated products or intentional ingestion. There is documentation that rodenticide can also be absorbed transcutaneously and via inhalation [2]. One study of 24 patients found that patients with rat poisoning did not know they had been exposed to rodenticide [7]. Due to this, clinical suspicion should be high when laboratory workup is suggestive of rodenticide poisoning but no known exposure is readily identified.

Rodenticides are classified based on type. There are LAARs, such as brodifacoum and difenacoum [2]. There is a neurotoxic agent, bromethalin, that works by uncoupling mitochondrial oxidative phosphorylation [8]. There are also illegal rodenticides containing acetylcholinesterase inhibitors [9]. The mechanism of action of LAARs is the inhibition of vitamin K epoxide reductase. This results in a deficiency of active vitamin K-dependent clotting factors [2].

Individuals with LAAR poisoning have varying degrees of severity of disease and varying presentation. Symptoms in adults are typically more severe than in children due to both ingestion of larger quantities of poison and preexisting comorbidities [2]. Children commonly experience mild coagulopathy, while adults have more severe coagulopathy, including mucocutaneous, urinary tract, and gastrointestinal bleeding. They can also experience intracranial hemorrhage, which is the most common cause of death from rodenticide poisoning. Approximately 2% of all exposures result in morbidity and mortality [2].

Treatment of rodenticide poisoning consists of giving intravenous vitamin K to aggressively reverse the coagulopathy. The initial dose is 50–100 mg, which is based on the severity of coagulopathy. Given the long half-life of rodenticides, not only do patients need long-term monitoring but also need long-term oral vitamin K therapy. One study observed that the average duration of vitamin K therapy for patients with rodenticide poisoning was 168 days [2]. Once there is suspicion of LAAR poisoning, high-dose vitamin K should be given empirically, prior to confirmation of the diagnosis [7]. One study of 32 patients found that those prescribed vitamin K at discharge had fewer readmissions [1]. Occasionally, recombinant FVIIa and prothrombin complex concentrate can be utilized to stop life-threatening bleeds [7, 10]. Long-term administration of vitamin K in the setting of rodenticide poisoning has complications. It is not uncommon to experience hypersensitivity reactions or infusion reactions to the intravenous formulation. Patients can also experience gastrointestinal symptoms such as nausea, vomiting, and diarrhea, limiting compliance [2, 11]. In addition to serial monitoring of prothrombin and activated partial thromboplastin times, some providers also repeat blood LAAR testing to guide the ultimate duration of therapy.

This case was notable for a lack of known ingestion or exposure to rodenticide after careful consideration of home or workplace rodenticide use, suicide attempt, or intentional poisoning, leading to the conclusion that ingestion occurred due to consumption of rodenticide-bearing animals. We speculate that a number of social and biological factors may have contributed to this patient's rodenticide poisoning. First, there is a local practice of farming “bamboo rats” (*Rhizomys sinensis*) for consumption that is already associated with transmission of a number of infectious diseases [12]. Second, there is widespread use of rodenticides in China that has previously led to epidemics of human illness, such as the neurologic syndrome, coma, and death caused by tetramine [13]. Third, consumption of predator species could enable exposure to even higher levels of rodenticide than consumption of rodents. Rodenticides can bioaccumulate in food chains and have been reported at high levels in a variety of predator species, such as red fox and raccoon in Finland, predatory birds and carnivores Spain, and dugites, bobtails, and tiger snakes in Western Australia [14–16]. Given the variety of animals the patient consumed, his LAAR toxicity may not be due to consumption of rodents but an animal that preys on rodents. Since rodent consumption remains a global phenomenon found across geographic and cultural divides, we argue that a careful dietary history is relevant to the workup of patients with suspected superwarfarin poisoning.

## 4. Conclusion

The incidence of rat poisoning in adults is low, and diagnosis is limited by two factors: clinical suspicion and the detection limits of laboratory tests. In these cases, a broad differential must be considered. A thorough history is critical, as many cases of rat poisoning in adults are either intentional or secondary to contamination. Clinicians should work closely with their laboratories to ensure that accurate results are obtained. It is important to note that depending on the amount of rodenticide ingested and the severity of coagulopathy, it may take several doses of high-dose vitamin K before normalization of coagulation labs is seen. The patients should be followed closely as an outpatient, as the coagulopathy may persist for several months after the initial ingestion, with regular coagulation labs until labs remain persistently normal.

## Data Availability

Data sharing is not applicable to this article as no datasets were generated or analyzed during the current study.
